# Transcriptome analysis of grain development in hexaploid wheat

**DOI:** 10.1186/1471-2164-9-121

**Published:** 2008-03-06

**Authors:** Yongfang Wan, Rebecca L Poole, Alison K Huttly, Claudia Toscano-Underwood, Kevin Feeney, Sue Welham, Mike J Gooding, Clare Mills, Keith J Edwards, Peter R Shewry, Rowan AC Mitchell

**Affiliations:** 1 Rothamsted Research, Harpenden, Hertfordshire, UK; 2 School of Biological Sciences, University of Bristol, Woodland Road, Bristol, UK; 3 School of Agriculture Policy & Development, University of Reading, Earley Gate, Reading, Berkshire, UK; 4 Institute for Food Research, Norwich Business Park, Colney, Norwich, UK

## Abstract

**Background:**

Hexaploid wheat is one of the most important cereal crops for human nutrition. Molecular understanding of the biology of the developing grain will assist the improvement of yield and quality traits for different environments. High quality transcriptomics is a powerful method to increase this understanding.

**Results:**

The transcriptome of developing caryopses from hexaploid wheat (*Triticum aestivum*, cv. Hereward) was determined using Affymetrix wheat GeneChip^® ^oligonucleotide arrays which have probes for 55,052 transcripts. Of these, 14,550 showed significant differential regulation in the period between 6 and 42 days after anthesis (daa). Large changes in transcript abundance were observed which were categorised into distinct phases of differentiation (6–10 daa), grain fill (12–21 daa) and desiccation/maturation (28–42 daa) and were associated with specific tissues and processes. A similar experiment on developing caryopses grown with dry and/or hot environmental treatments was also analysed, using the profiles established in the first experiment to show that most environmental treatment effects on transcription were due to acceleration of development, but that a few transcripts were specifically affected. Transcript abundance profiles in both experiments for nine selected known and putative wheat transcription factors were independently confirmed by real time RT-PCR. These expression profiles confirm or extend our knowledge of the roles of the known transcription factors and suggest roles for the unknown ones.

**Conclusion:**

This transcriptome data will provide a valuable resource for molecular studies on wheat grain. It has been demonstrated how it can be used to distinguish general developmental shifts from specific effects of treatments on gene expression and to diagnose the probable tissue specificity and role of transcription factors.

## Background

Cereals are of immense importance to humankind with over 2000 million tonnes being harvested annually and used for food, livestock feed and industrial raw materials. These uses exploit the reserves of starch and protein, which are deposited in the endosperm which accounts for about 80% of the mature grain. Hence, grain yield and end use quality are largely determined by thesize and composition of the endosperm.

The endosperm is formed by a second fertilisation within the embryo sac, with two central cell nuclei and one pollen nucleus fusing to give a triploid constitution. Subsequent cereal endosperm development can be divided into a number of stages [1]. The first of these is free nuclear division which occurs within the primary endosperm cell to give a coenocyte which, in wheat, may contain over 2,000 nuclei by 72 hours after fertilisation [2]. Cellularisation then occurs over a period of about 24 hours, followed by a period of about 10 days during which cell division, expansion and differentiation occur to give the characteristic structure of the endosperm with a total of up to 300,000 cells [1,2].

A major transition point occurs at about 14 days after fertilisation in wheat grown in temperate climates, marking essentially the end of endosperm cell division [1] and the start of grain filling (the deposition of starch and gluten proteins) in these cells. After about 28 days the deposition of storage reserves decreases and the grain starts to desiccate, reaching physiological maturity at about 42 days and harvest ripeness 1–2 weeks after this. However, the duration of these phases differ greatly between climates with the maximum dry weight being achieved by approximately 21 days in N. America [3].

Transcriptomics have been used to relate transcript abundance to these changes in developing wheat and barley grain. Microarrays of wheat cDNA [4,5] and a macroarray of barley cDNA elements [6] have been used to follow selected parts of the transcriptome. Alternatively, opensystems based on counts of sequences have been applied by Kawaura *et al*. [7] who classified the expression patterns of two groups of storage protein genes from EST abundances and McIntosh *et al*. [8]who used Serial Analysis of Gene Expression (SAGE) on developing wheat grain. The two approaches are complimentary; arrays allow greater resolution of expression differences and ease of comparison from a fixed platform, whereas sequencing approaches allow discovery of novel transcripts.

Although cDNA-based arrays provide valuable information they give only partial coverage of the genome, for example, Laudencia-Chingcuanco *et al*. [4] used a cDNA array of 7,835 elements but the total number of genes in hexaploid bread wheat probably exceeds 100,000. The wheat Affymetrix GeneChip^® ^array comprises over 61,000 sets of eleven 25 mers ('probesets') representing 55,000 wheat transcripts and may cover half of the wheat expressed genes. This platform has been used to study the transcriptomics of meiosis in wheat [9] and, in the first e-QTL study in wheat, to identify loci controlling seed development [10]. Affymetrix arrays have significant advantages over cDNA arrays in terms of data quality and ease of comparison between samples. In particular, it is known that the homoeologous genes from the three genomes of wheat can be expressed with different spatial and temporal specificities [11]; while cDNA array elements would be expected to cross-hybridise with these different transcripts, the multiple, short probes of the Affymetrix platform could in principle distinguish them [12]. We have therefore used this new resource in order to identify transcripts associated with grain development and filling in wheat.

Grain development is associated with massive changes in gene expression andany comparisons between genotypes or environments therefore needs to place the results in a developmental context. The data reported here constitute a reference data set to allow this as illustrated with an experiment on effects of heat and drought treatments on the wheat grain transcriptome. The dataset can also be exploited for further studies of grain development; here we have chosen to focus on transcription factors as key regulators of transcription.

## Results and discussion

The selected winter wheat cultivar, Hereward, was released in the UK in 1991, but is still widely grown and has remained a "gold standard" for breadmaking wheats.

### Grain development of cv. Hereward

**Figure 1:**
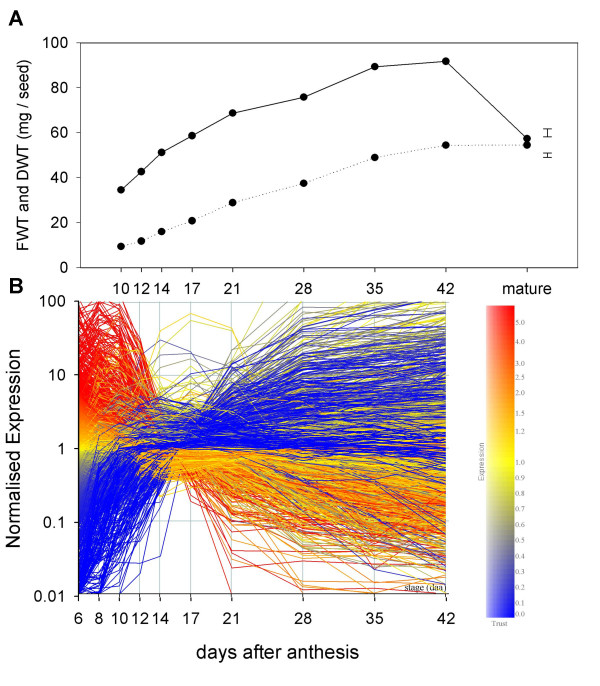
**Data from two biological replicate samples of developing wheat caryopses.** Upper panel: grain fresh weight and dry weight (error bars are least significant difference at P < 0.05 from 1-way analysis of variance). Lower panel: transcriptome of samples measured on wheat Affymetrix arrays. Each line depicts average signal from two replicate samples for a probeset coloured according to expression at 6 days after anthesis (daa). Gene set is those showing significant (P < 0.05; Benjamini-Hochberg false discovery rate multiple-testing correction) differences in expression between stages (14,550 probesets).

**Figure 2:**
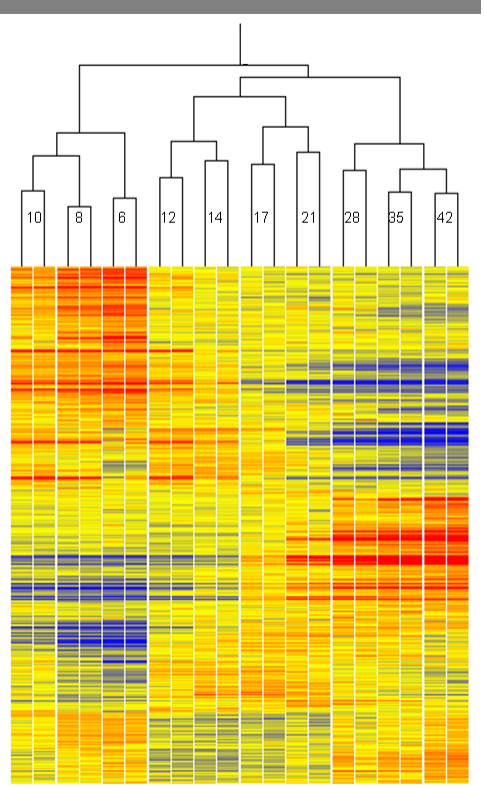
**Hierarchical clustering of samples by gene expression.** Gene set is same as that in Figure 1. Co-expression measure was Pearson correlation. All replicates cluster as pairs, for which the stage is shown as days after anthesis.

Approximately 33,000 of the 61,000 probesets on the array showed significant binding to transcripts and, of these, 14,550 showed significant differences in transcript abundance between developmental stages. (Note: Transcriptomics is the measurement of transcript abundance, but here we follow common practice using the terms 'gene expression' and 'transcript abundance' interchangeably, so that both include variation in transcript degradation). The profiles of this latter set during grain development are summarised with the changes in seed dry and fresh weights in Figure 1. Hierarchical cluster analysis of the whole dataset (Figure 2) shows that biological replicate samples cluster together, with successive changes in the patterns from 6 to 42 daa. Furthermore, three broad phases are indicated, with the samples from 6, 8 and 10 days, 12, 14, 17 and 21 days, and 28, 35 and 42 days forming separate clusters.

These phases can also be seen in the highly distinctive pattern of normalised transcript abundance (Figure 1B) which shows massive changes in expression for many genes in the switch from 10–12 days and again from 21 to 28 days. Similar changes can be seen in the smaller set (2,237) of differentially regulated transcripts identified by cDNA arrays [4] and in the profiles of the 250 most abundant SAGE tags [8].

**Figure 3:**
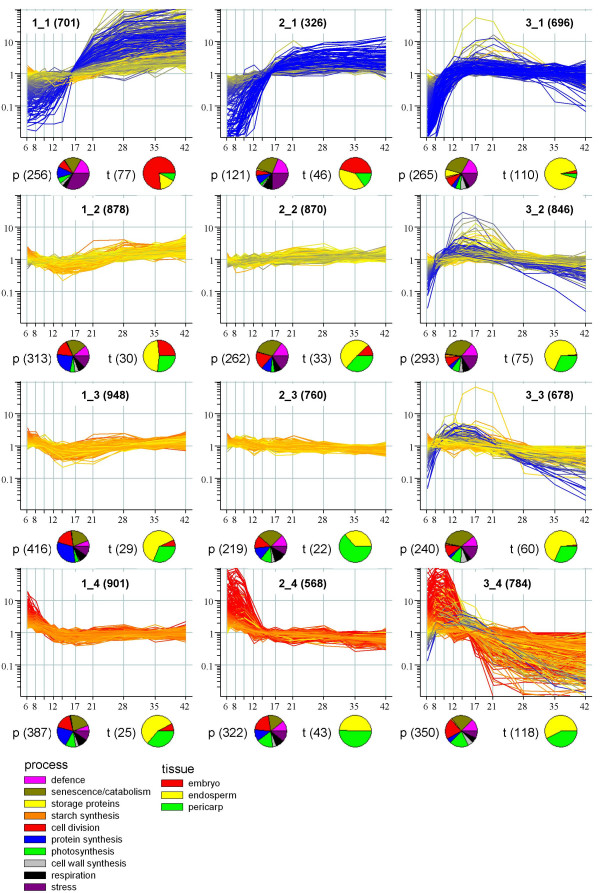
**Day of anthesis versus normalised expression of probesets grouped into clusters according to their expression profiles by Self-Organising Map algorithm.** Each graph represents a different cluster and at the top of the graph the cluster name (which is the position in the map) is shown along with the number of probesets it contains in parentheses. Pie chart classifications for biological process and tissue category were inferred from various sources (see Methods). Unclassified probesets were omitted from the pie charts: the number that were classified are shown in parentheses next to the chart.

It is useful to aggregate genes with similar expression profiles to find if probesets with particular properties are over-represented in these clusters. The dataset comprised hundreds of statistically significant different gene expression profiles, but for display purposes we chose to aggregate these into the 12 sets shown in Figure 3 using the Self-Organising Map algorithm. This is appropriate for an overview display as it places similar gene clusters next to each other, so each dimension represents a progressive change in the expression profile of the cluster.

In order to associate the gene clusters with biological processes, we assigned probesets to ten process categories chosen to be of most relevance to grain development (see key of Figure 3; and Methods). Whereas most probesets could be identified in terms of their molecular function (e.g. transcription factor, protein kinase), only 38% of them could reliably be associated with these processes. The number of probesets in each category are summarised for each cluster in the left hand pie charts shown in Figure 3. Whole developing caryopses comprise three main types of tissue: the endosperm, the embryo and the outer, maternal (mainly pericarp) tissues. We therefore assigned putative tissue locations to 668 of the transcripts based on published biochemical studies of the encoded proteins and on the locations reported in the barley transcriptome study of Sreenivasulu *et al*. [6], who analysed separate pericarp, endosperm and embryo tissues. Additional information on tissue locations came from the *in situ *hybridisation database of Drea *et al*. [13]. The putative locations of the transcripts in each set are summarised in the right hand pie charts in Figure 3. The assignment of probesets to process and tissue classifications are available [see Additional file 1].

Based on their putative assignments of function and tissue location it is possible to relate the changes in gene expression profiles to stages of grain development. An overall pattern is immediately apparent with embryo transcripts tending to increase throughout development (clusters shown on top left of map in Fig. 3, i.e. 1_1, 2_1, 1_2), endosperm transcripts tending to increase to a plateau starting at 14 daa (top right) and some endosperm and pericarp transcripts decreasing through development (bottom right).

The cellularisation of the coenocytic endosperm is usually complete between 6 and 8 days after anthesis and is followed by a period of active cell division, expansion and differentiation to establish the starchy endosperm and aleurone tissues. The embryo develops more slowly than the endosperm duringthis period while the pericarp remains metabolically active. This phase corresponds to the 6, 8 and 10 day samples in our analysis and many of the transcripts which are expressed most highly during this earliest period (Figure 3 1_4, 2_4, 3_4) are associated with the endosperm and pericarp and with cell division, photosynthesis and development rather than storage product (starch and protein) synthesis.

Grain filling is initiated at about 10 daa and continues until about 28 days. This is associated with very high abundance of specific transcripts (2_1, 3_1, 3_2) but these are only represented by about 50 distinct probesets. As a result, and because the data shown in Figure 3 are normalised to median gene expression for display purposes, the dominance of these transcripts during grain fill is not apparent. However, this is clear when our data are expressed on an absolute basis (not shown) and confirms results from other transcriptomics approaches (cDNA arrays, SAGE tag and EST counts), which show storage protein transcripts to be the most abundant in developing seeds of wheat [4,8] and rice [14]. These transcripts tend to reach a maximal level at around 14 daa which is maintained (relative to the total transcriptome) until 42 daa.

Many transcripts associated with the pericarp and photosynthesis decline steadily from the start of the sampling (1_4, 2_4, 3_4); however others maintain a more constant level of expression throughout the developmental series (1_3, 2_3).

In contrast, the majority of embryo transcripts continue to rise until the end of the sampling period (42 days). Transcripts expressed highly during this latter period include many related to defence and stress (1_1, 2_1), in agreement with SAGE results [8]. The stress transcripts may relate to embryo desiccation; for example, dehydrins are exclusively in group1_1. During this same period (28–42 days) there are decreases in transcripts associated with the endosperm and pericarp (3_3, 3_4).

Several clusters show more subtle, albeit significant, changes in expression throughout development associated with all three tissues (1_2, 1_3, 2_2) or with the endosperm and pericarp (2_3). These four clusters include many transcripts encoding proteins expected to be present in almost every cell type, e.g. mitochondrial proteins, machinery for protein synthesis and degradation, enzymes of primary metabolism.

All clusters contain a small proportion (1–2%) of probesets that are in the antisense orientation when compared to coding rice sequences. These presumably function to down-regulate the sense transcript *in vivo*, many of which seem to be involved in protein synthesis and degradation (ribosomal proteins, proteases, ubiquitin, proteasome). A similar fraction of transcripts was identified as being antisense using SAGE technology on developing wheat grain [8].

The clusters identified here can be compared with those reported in other transcriptome analyses of developing wheat [4,8] and barley [6] grain by identifying similar sequences [see Additional file 2]. The separation into a small number of gene clusters is to some extent arbitrary and dependent on choice of algorithm; nevertheless some trends are clear, e.g. cluster 3_1 is very similar to McIntosh et al. cluster 2j (19 out of 20 matching sequences), Sreenivasulu *et al*. cluster 5,3 and Laudencia-Chingcuanco *et al*. cluster 6. Those clusters that have similarities in sequence composition [see Additional file 2], also show similar average expression profiles. This shows some conservation of effects across conditions and between wheat and barley. However, it is noticeable that the size of changes in apparent transcript abundance observed here are often greater than in these other experiments. Possibly the EST-based platforms tend to integrate across several similar transcripts thus giving a damped signal compared with the oligonucleotide-based Affymetrix platform [12].

### Effects of environmental factors on grain development

Environmental factors are known to have effects on wheat grain development, with impacts on both yield and end-use quality [15,16]. The effects of heat, drought and heat & drought on the grain transcriptome profile of cv. Hereward, were therefore studied, selecting a highest temperature of 28°C. This temperature is sufficient to affect yield and quality [17] but substantially below temperatures which are known to affect wheat storage protein gene expression [18]. The plants were grownin controlled environment (CE) cabinets and subjected to different conditions from 14 daa.

**Figure 4:**
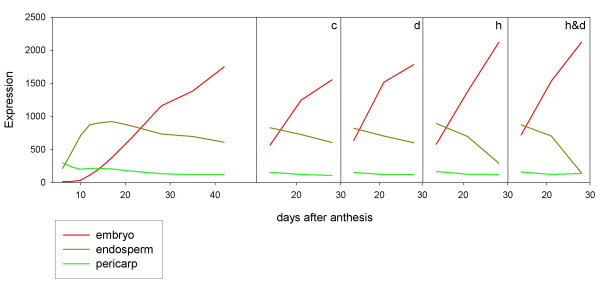
**Geometric average of expression for all probesets likely to be predominantly expressed in embryo, endosperm or pericarp tissues for both developmental series (left panel) and controlled environment experiment with control (c), drought (d), heat (h) and heat & drought (h&d) treatments (right panels).** Embryo, endosperm and pericarp sets contain 104, 496 and 296 probesets, respectively.

**Figure 5:**
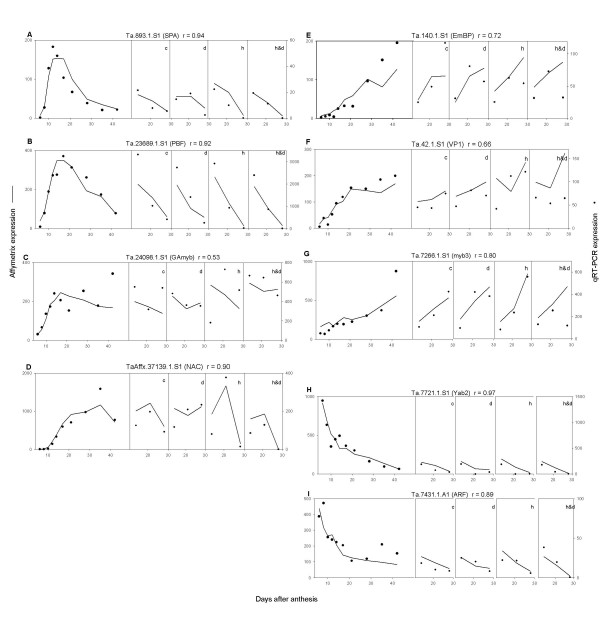
**Comparison of expression estimates from wheat Affymetrix array (lines) and quantitative reverse transcription PCR (qRT-PCR; points) for nine transcription factors.** The left hand panel displays data from the developmental series which are the average of two replicates. The other panels are the unreplicated controlled-environment data for control, dry, hot and hot & dry conditions. Correlation coefficients (r) between the two expression measures are indicated.

**Figure 6:**
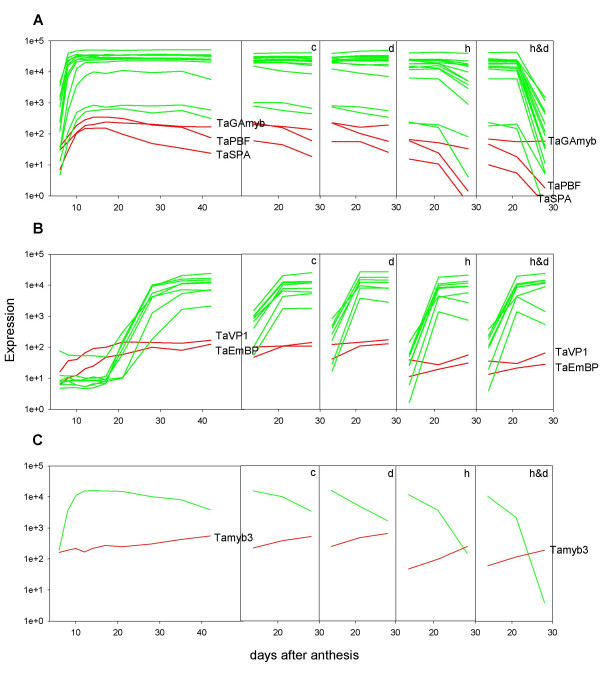
**Comparison between expression of transcription factors (red lines) and their known targets (green lines).** A: Transcripts of the TaSPA, TaPBF and TaGAmyb transcription factors and LMW glutenins. B: Transcripts of TaEmBP and TaVP1 transcription factors and Em proteins. C: Transcripts of HvMYB3-like transcription factor and BTI-CMe trypsin inhibitor protein.

**Figure 7:**
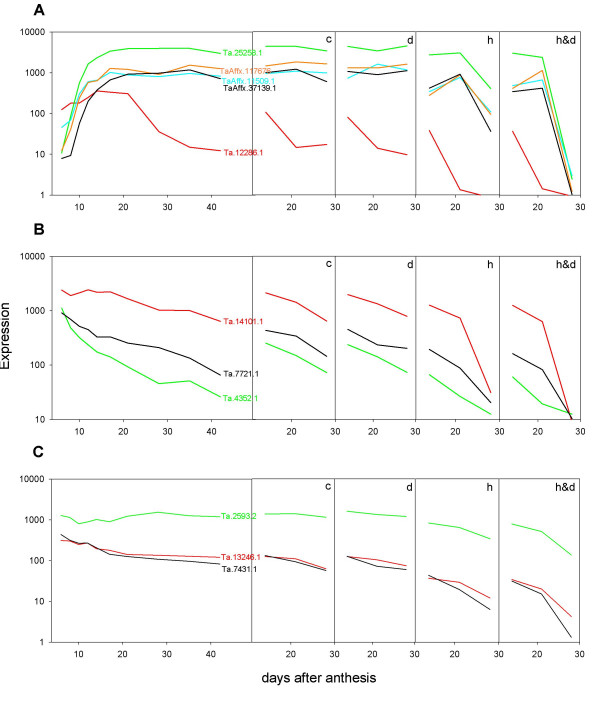
Expression profiles for probesets matching putative NAC (A), YABBY (B) and ARF (C) transcription factors.

The CE datasets show the same trends as observed in the developmental series but are accelerated, especially in hot and hot & dry conditions, as shown by the average expression of gene clusters [see Additional file 3] or by gene sets where the likely tissue of expression is known (Figure 4). (Note:the expression measure shown in Figs. 4, 5, 6, 7, whilst not normalised to median gene expression, is still normalised to the total transcriptome at each time point; if expression were to be calculated on a per caryopsis basis the values would be much lower later in development as the total amount of RNA decreases.) Thus the abundances of embryo-associated transcripts increase throughout while those of endosperm-associated transcripts increase to a plateau. These changes occur faster in the heat treated samples and the late decreases in endosperm associated transcripts are greatly exaggerated in the 28 daa heat treated samples (Figure 4; Additional file 3 3_2). The pericarp-associated transcripts decreased steeply till 10 daa then more gradually (Figure 4).

These environmental effects on development are expected since temperature is known to accelerate development and measures such as thermal time have been used in order to quantitise this effect. Drought can also accelerate grain development due to stomatal closure, which reduces transpirational cooling, and due to increases in the rate of desiccation. It is possible to accurately quantify these effects on the transcriptome from the array data by estimating the equivalent stage in the developmental series for each of the CE experiment samples. This was done by calculating a distance measure between the CE samples and the interpolated developmental series [see Methods and Additional file 4]. The value of daa which gives the minimum distance is an estimate of the developmental stage of the 12 samples (Table 1). These estimates are not sensitive to changes in the sample of probesets used, as shown by the results of a bootstrapping procedure.

**Table 1:** Estimates of equivalent developmental stage, derived from comparison of transcriptomes, expressed as daa in the developmental series for 12 similar samples measured in a different experiment. The estimates are shown and also all the estimates from 200 bootstrapped re-samplings of the 32,512 probesets with the frequency indicated in parentheses (see Methods).

	**14 daa**		**21 daa**		**28 daa**
**CE samples**	**estimate**	**bootstrap**	**estimate**	**bootstrap**	**estimate**

**control**	22	22 (200)	34	33 (97) 34 (103)	41
**dry**	23	23 (200)	38	38 (200)	>42
**hot**	20	20 (200)	37	37 (200)	>42
**hot & dry**	22	22 (192) 23 (8)	39	39 (195) 40 (5)	>42

This analysis showed that the developmental stage of CE samples at 14 daa (the start of the imposition of different environmental conditions) is equivalent to about 20–23 daa in the developmental series; this is expected as the temperature regime used was 23°C/15°C day/night compared to 18°C/15°C in the developmental series. The variation in equivalent daa probably reflects differences between the separate cabinets used.

Increasing the temperature to 28°C/20°C day/night or reducing the water to 44% field capacity both accelerated development over the first seven days of treatment as the control samples progressed the equivalent of 12 days, the dry 15 days, the hot 17 days and the hot & dry 17 days. After 14 days treatment (i.e. at 28 daa) the control sample was equivalent to 41 daa and the other three samples had progressed beyond the final sample taken in the developmental series (at 42 daa). The trends [see Additional file 4 Panel C] show that the treatments continued to accelerate development, with the size of effect being dry < hot < hot & dry.

Altenbach and Kothari [19] showed that the effect of temperature on expression of some selected genes in wheat grain was consistent with the acceleration of physiological markers of development. Our estimates of developmental stage from the transcriptome agree well with those using the moisturecontent of the grain, if the values for the CE samples [17] are compared with those from the developmental series (not shown). This suggests that water status acts as a major signal for control of wheat caryopsis development (also postulated by McIntosh *et al*. [8]).

Using this transcriptome analysis, it is also possible to identify genes which are affected by the environmental treatments independently of general developmental effects. The expression values from the CE samples at 21 daa were corrected to the closest developmental stage from Table 1. Probesets were selected which were more than two-fold changed in this corrected expression for both drought-treated or both heat-treated samples relative to corresponding samples lacking these treatments (Table 2). Transcripts specifically up-regulated due to drought seem to have a role in non-starch polysaccharide hydrolysis, whereas those down-regulated include a Lt1.1 transcript; homologues of Lt1.1 have been shown to be highly responsive to environmentalconditions, being induced by low temperature (e.g. [20]). The most highly up-regulated transcript under heat is Rubisco activase whichhas been shown to be inducible by heat and is consistent with its role in maintaining Rubisco integrity at high temperature [21]. Surprisingly, a transcript for a heat shock protein appeared to be down-regulated in the heat-treated samples. Altenbach and Kothari [19] also identified a few transcripts as affected by temperature, independently of developmental effects, but these effects were not seen here, probably because of the more moderate high temperature treatment (28/20 compared to 37/28°C). Overall, acceleration of development explained the great majority of the changesobserved at 21 daa; 93% being within a factor of 1.5 of the predicted value.

**Table 2:** Probesets identified as being early responsive to environmental treatments, when corrected for general developmental effects. The corrected effects, expressed as transcript abundance ratios, of drought (average of drought/control and heat & drought/heat) or heat (average of heat/control and heat & drought/drought) are shown. The probesets shown had at least a two-fold change in both the averaged comparisons.

**DROUGHT UP**	**corrected drought effect**	**descriptor**
TaAffx.92642.1.A1	13.4	putative polygalacturonase precursor
TaAffx.109111.1.S1_a	13.2	putative polygalacturonase precursor
Ta.10.1.S1_a	5.7	1,3 beta glucanase
Ta.18596.1.S1	6.6	similar beta-expansin 1a precursor
Ta.25483.1.S1	4.6	similar putative nodulin 3
Ta.13457.1.S1	4.1	unknown
TaAffx.56781.1.S1	3.8	unknown
**DROUGHT DOWN**		
Ta.23822.1.S1	0.2	Cytochrome P450 71C4
Ta.7479.1.S1_a	0.3	Lt1.1 protein
Ta.3380.1.S1	0.3	Cytochrome P450 (CYP71C3v2-like)
**HEAT UP**	**corrected heat effect**	
Ta.1404.1.S1	5.1	ribulose bisphosphate carboxylase activase B
Ta.25880.1.A1	3.4	unknown
**HEAT DOWN**		
TaAffx.42798.1.A1	0.3	similar cysteine proteinase EP-B1 precursor
Ta.12225.2.S1	0.3	similar ethylene-responsive transcriptional coactivator
Ta.202.1.S1	0.3	heat shock protein 26.6B
TaAffx.143996.1.S1_s	0.3	similar Histone H4
Ta.9600.1.S1_x	0.3	similar early light-inducible protein

### Expression of transcription factors

The wheat Affymetrix GeneChip^® ^contains about 2,000 probesets for potential transcription factors (TFs). Relatively few TFs have been characterised in cereals and even fewer of their target genes and/or biological roles determined. Available evidence, however, suggests that TFs and their targets display a many-to-many relationship: thus, multiple TFs bind to a promoter while individual TFs control multiple genes. Transcription is either controlled through the requirement for a set of factors to be present in sufficiently high numbers in the right cells at the right time, or via TF modification to alter their binding to DNA or each other. The first model predicts the differential expression of factors together with the genes they control; the second does not. The expression of around a half of the total TFs interrogated was constant during grain development while the remainder were distributed between the expression profiles groups outlined. We selected nine TFs for detailed analysis, using qRT-PCR to confirm the changes in expression levels determined using the GeneChip^® ^arrays (Figure 5),

The relative expression levels determined by qRT-PCR showed excellent agreement with those determined using arrays, with correlation coefficients ranging from 0.89 to 0.97 in five out of the nine cases, while the other four varied between 0.79 and 0.53. This degree of agreement is very close to that observed for other genes by [9].

The expression profiles of the TFs selected showed three distinct patterns, associated with gene expression predicted to be in the endosperm, embryo or pericarp (Figure 4).

#### Endosperm-like expression

Four of the TFs (Figure 5A, B, C, D) displayed an endosperm expression pattern typical of 2_1, 3_1, 3_2 (Fig. 3). The heat and heat & drought treatments precipitated the decline of the transcripts presumably in line with accelerated maturation of the endosperm.

##### TaSPA and TaPBF

Many prolamin storage protein genes (including those encoding α-gliadins and low molecular weight (LMW) subunits) contain the cis-element known asthe endosperm box in their promoters. This bipartite element is composed ofthe GCN4 box to which the bZIP TFs TaSPA (wheat), and BlZ2 (barley) bind and the prolamin box to which the DOF TFs WPBF(TaPBF) and BPBF bind [22-25]. Previous Northern analysis suggested that TaSPA and TaPBF were endosperm-specific with transcript levels peaking around 15–18 daa. Our data(Figure 5A &5B) is in good agreement with this as their expression parallels the rise in transcripts for LMW subunit and α-gliadins genes (Figure 6A) which is consistent with a role in prolamin gene expression. Additional targets are likely to fall into the same grain filling expression profile, such as the trypsin inhibitor BTi-CMe genes (Figure 6C), known targets of BPBF and BIZ2 [26]. However, not all prolamin genes have an endosperm box [27]. The high molecular weight (HMW) subunit genes, for example, presumably rely on other TFs potentially with similar profiles to TaSPA and TaPBF: 54 other putative TFs from 13 families are represented in the predominantly endosperm 3_1 cluster.

##### TaGAmyb

The R2R3 class barley HvGAmyb TF, initially isolated based on its ability to bind to GA responsive α-amylase promoters expressed in the aleurone during germination also binds to the AACA elements of the B hordein (*Hor 2*) and BTI-CMe (*iTrr-1*) promoters, and interacts with BPBF [28]. Their Northern data showed moderate levels of HvGamyb in the endosperm from 10 to 22 daa, while *in situs *at 20 daa indicated expression tobe mainly in the aleurone layer and embryo. TaGAmyb has a pattern of expression (Figure 5C), typical of group 2_1, which would be consistent with roles in endosperm grain filling and in the embryo. α-Amylase genes are not normally expressed in late grain development but in some genotypes and under specific environmental conditions pre-harvest sprouting or premature amylase production can occur [29]. High levels of TaGAmyb late in grain development may contribute to this phenomenon.

##### TaNAC family

The TF encoded by Ta.37139.1 has homology to the NAC class of TFs [see Additional file 5]. This large plant-specific TF family (123 in rice PlnTFDB; [30]) contain a NAM DNA domain that binds to the core sequence CACG. Their members regulate developmental processes, as well as defence and abiotic stress responses [31]. Although still consistent with endosperm expression, transcripts corresponding to Ta.37139.1.S1 peaked later in grain development compared to the expression of TaPBF2 and TaSPA.

Several other NAC TFs were also included in the array (Fig 7a). Although their expression pattern was not verified by q-RTPCR, those represented by probe sets Ta.11509.1.S1, TaAffx.117676.1.S1 and Ta.25258.1.S1 showed very similar expression patterns to Ta.37139.1.S1 and their encoded proteins sharea very high degree of sequence similarity with each other and to several barley endosperm expressed NAC genes [6]. Phyologenetic analysis [see Additional file 6] of the NAC related sequences from the TIGR gene indices and PlnTFDB database, placed the wheat and barley genes in a small subcladeof the NAM group [32] together with two TFs from rice and the maize *nrp1 *and *ZmNAC4 *genes. Consistent with our data, expression of the maize genes is confined to starchy-endosperm cells [33,34], while MPSS data for the two rice genes suggests their expression is confined to the developing grain. No functions have been ascribed to these proteins; however, their tissue specificity and the fact that the maize *nrp1 *gene is a maternally controlled imprinted gene, may indicate an important role in endosperm development.

Interestingly, the gene represented by probe set Ta.12286.1.A1, had a distinctly different expression pattern (Figure 7A) although was still highly homologous to the others [see Additional file 5], suggesting either this protein has a different function, or performs the same function in other tissues of the developing grain.

#### Embryo-like expression

Three of the other TFs chosen for further analysis showed an embryo like expression pattern similar to group 1_1 and 2_1 transcripts.

##### TaEmBP and TaVP1

TaEmBP a bZIP TF [35] and ZmVP1 an ABI3B3 class TF [36] are known to be associated with maturation of the embryo and aleurone layer and the expression profiles of TaEmBP and TaVP1 both showed an embryo like pattern (Figure 5E, F). Known targets of both factors include genes encoding the Em (early methionine) protein that are involved in protecting cells against tissue damage during seed desiccation [37]. Expression of the multiple Em genes is induced by abscisic acid (ABA) and involves both EmBP and VP1, binding to the G-box in the abscisic acid response element (ABRE[38]). Expression of the Em genes present in the array (Figure 6B) was typical of group 1_1 transcripts, thus consistent with TaEmBP and TaVP1, being responsible for their expression.

Maize VP1 is also known to be involved in expression of the aleurone specific myb TF gene C1 and in the repression of α-amylase gene expression in the aleurone layer late in grain development [39,40]. Accordingly, ZmVP1 was found to be highly expressed in both developing embryos and the aleurone layer. Since Em mRNA accumulates to high levels in wheat aleurone cells (unpublished) it is likely that this is also true in wheat which would mean group 1_1 contains transcripts expressed in both embryo and aleurone.

##### TaMyb3

HvMyb3 a myb-related (SHAQKYF R1myb) TF was reported to be capable of interacting with BPBF and BLZ2 and to bind to the TATC elements in the promotersof the *Itr-1 *(BTI-CMe), and α-amylase *Amy6.4 *genes [26]. The expression profile of the wheat TaMyb3 (Ta.7266.1.S1) orthologue (Figure 5G), however, does not reflect those of BTI-CMe (Figure 6C), or the LMW subunit genes (Figure 6A), nor TaGAmyb (Figure 5C), which potentially regulates the same spectrum of genes, and is more consistent with a primary role in the embryo for this factor. In fact HvMyb3 was also shown to be expressed in barley embryos in addition to the developing endosperm, but it ispossible that the roles of HvMyb3 and TaMyb3 may have diverged.

#### Pericarp-like expression

Two previously uncharacterised TFs showed a typical pericarp like expression similar to transcripts in groups 2_4 and 3_4 (Figure 2).

##### TaYab2

The protein corresponding to probe set Ta7721.1.S1 (Figure 5H) has homologyto the class of TFs known as C2C2-YABBY, all of which contain a zinc-fingerDNA binding domain and a HLH YABBY domain. This small plant-specific TF family contains seven to eight members in rice and six in Arabidopsis, where they have been shown to be involved in establishing abaxial-adaxial polarityin lateral organs and in restricting meristem initiation and growth [41]. Characterisation of the genes in monocots is less advanced, but mutational and expression analysis suggest that their functions have diverged between monocots and dicots, with the monocot TFs lacking a central role in specifying abaxial-adaxial cell fate [42].

Phylogenetic analysis [see Additional file 6] shows that apart from Ta7721.1.S1 the wheat Affymetrix chip also has probesets for wheat homologues to all of the rice genes [43] apart from OsYab1 and OsYab7. The wheat Ta *yab*3, 4, and 5 genes are not expressed in developing grain (which is also true of their counterparts in rice), while Tayab2 (Ta7721.1.S1), *Ta*DL (Ta4352.1.S1), and Tayab6 (Ta.14101.1.S1) all showed broadly similar pericarp like patterns of expression (Figure 7B); which is consistent with the pericarp expression reported for HvDL and early grain development forOsyab2, 6 and OsDL [6,43]. Our data are consistent with a role for all these yabby proteins in pericarp development in wheat.

##### TaARF

Probeset Ta.7431.1.A1 (Figure 5I), shows homology to the auxin response factor (ARF) family of TFs that bind specifically to TGTCTC-containing auxin response elements (AuxREs). This relatively small TF family (25 members in rice) play a pivotal role in auxin-regulated gene expression of primary response genes [44]. The wheat gene sequence is most closely related to the rice OsARF22 and the Arabidopsis *AtARF16 *and *10 *genes [see Additional file 7] that are highly expressed in most tissues [45]. The function of these ARFs is unknown but the wheat gene expression profile would be consistent with an auxin-mediated role in pericarp development. A second ARF represented by the probe set Ta2593.2.S1 was also highly expressed in developing grain although in a pattern consistent with roles in both the pericarp and endosperm (Fig. 7C), This gene is most closely related to OsARF4 of rice and At *ARF*2 of Arabidopsis. Mutants in *AtARF*2 result in pleiotropic effects related to its repression of cell division. For example, knockouts of *ATARF2 *lead to extra cell divisions in the integument, which in turn result in the production of larger seeds [46]. It would be of interest to determine if Ta2593.2.S1 has a similar role in the pericarp and endosperm of wheat.

## Conclusion

The transcriptome of developing wheat caryopses shows massive changes in transcript abundance which can be related to key processes driving development. The data presented here represent a resource which can be exploited by those studying transcriptomics in wheat grain to place their results in a developmental context, exemplified by analysis of the effects of heat and drought treatments. We have also shown that the time course data can be interpreted to provide evidence on the tissue specificity and putative function of transcription factors without the need to isolate individual tissues fromthe grain. It is likely that many future applied studies on wheat will also involve whole-grain transcriptomics on elite cultivars (as in a recent e-QTL study; [10]) so our data are suited to help interpret these. The value of this dataset will increase as knowledge of the function and spatial distribution of the transcripts represented on the Affymetrix wheat array improves. All the data has been submitted in MIAME-compliant form to the ArrayExpress database (Accession Number E-MEXP-1193).

## Methods

### Growth of wheat cv. Hereward

After 10 weeks vernalisation, plants were transferred to a glasshouse with day/night temperatures of 18°C/15°C and 16 hours light. The main stem heads were tagged at anthesis. Developing whole caryopses were then harvested 6, 8, 10, 12, 14, 17, 21, 28, 35 and 42 daa and immediately frozen at -80°C for RNA extraction. Each sample comprised approximately 100 caryopses taken from the central third of 10 heads, with two samples being taken from each developmental stage. These samples were treated as biological replicates for mRNA extraction and array analysis. A further 20 developing caryopses were harvested from the middle part of each ear at 10, 12, 14, 17, 21, 28, 35 and 42 daa and at maturity. Those were immediately frozen a -20°C for biochemical studies.

In the environmental-treatment experiment, plants were moved to controlled environment (CE) cabinets from the glasshouse at about 3 daa. They were initially grown with 70% relative humidity, day/night temperatures of 23°C/15°C and watering to 100% field capacity. Drought stress (44% field water capacity, 23°C/15°C day/night temperature), increased temperature (28°C/20°C, 100% field water capacity), and drought stress (44% field capacity) with increased temperature (28°C/20°C) stresses were then applied from 15 to 28 daa as described by [17]. Whole developing caryopses were collected at 14, 21 and 28 daa.

### RNA extraction

The method was modified from [47]. 1.5–3 g of tissue was ground in liquid nitrogen and were extracted in CTAB buffer (2% (w/v) CTAB, 2% (w/v) PVP K30, 100 mM Tris-HCl, pH8.0, 25 mM EDTA, 2.0 M NaCI, 0.5 g/l spermidine, 2% (w/v) β-mercaptoethanol). The supernatant was extracted twice with chloroform:IAA (24:1) to remove proteins. RNA was precipitated by addition of 0.25 vol. of 10 M LiCl and incubation on ice overnight. The RNA pellet was dissolved in SSTE buffer (1.0 M NaCl, 0.5% (w/v) SDS, 10 mM Tris HCl pH8.0, 1 mM EDTA) to remove polysaccharides and extracted once with chloroform:IAA. After ethanol precipitation, total RNA was dissolved in DEPC-treated water and stored at -80°C.

Total RNA was purified through RNeasy mini spin columns (Qiagen). About 2 μg of total RNA was loaded on MOPs gels to check the purity. The integrity of RNA was determined with an Agilent Bioanalyser 2000 CE system.

### Affymetrix Genechip^® ^hybridisation and data analysis

Transcriptional profiling was performed using the Affymetrix GeneChip^® ^Wheat Genome Array using the standard one-cycle cDNA synthesis protocol and hybridisation (as described in the GeneChip^® ^Expression Analysis Technical Manual). Transcriptome data analysis was carried out in the GeneSpring^® ^7 package (Agilent Technologies, Inc.) except where otherwise stated. Individual signals from the perfect match probes were pre-processed using the variant of the Robust Multichip Average algorithm which takes account of the probe GC composition [48]. Probesets were filtered to remove those (about 40%) which never showed expression values > 10 for any condition. The cross-gene error model option of GeneSpring was turned off so that only the true biological replicates were used to estimate variance. A one-way ANOVA was applied to the developmental series using Welch's t-test (which does not assume equal variance) with P < 0.05 and the Benjamini-Hochberg false discovery rate multiple testing correction. The 14,550 probesets that passed this test were deemed to show significant differential expression through development.

The condition tree in Figure 2 was derived from this gene set, using Pearson correlation as the co-expression measure. For cluster analysis, probesets where Affymetrix information indicated that they were likely to cross-hybridise were removed to give 8,956 probesets. Organisation of probesets into clusters with similar expression profiles was applied to this set using the Self Organising Map algorithm [49] with 100,000 iterations and radius = 6.0 to give 12 clusters arranged as a 3 × 4 map (Figure 3).

Probesets were allocated to tissue and biological process categories according to sequence similarity of the Affymetrix target sequence to known sequences. Tissue categorisation was based on (1) genes where tissue specificity of expression is known (e.g. glutenins), or (2) similarity (Blastn with E < 10^-10^) to barley ESTs identified as belonging to clusters which had tissue-specific expression by [6], or (3) similarity (>90% identity) to *in situ *probes which showed specific expression in wheat caryopsis sections [13]. The ten biological process categories were chosen to represent key processes in grain development. Probesets were allocated to these exclusively according to the occurrence of key terms in annotation retrieved from hits in Genbank nr database (Blastx with E < 10^-10^). Further allocations were made by retrieving the closest TIGR pseudomolecule (release 5) rice locus [50] for each probeset using the WhETS system [51]. The slimGO terms associated with each rice locus were then used to map process categories onto the probesets. The complete annotation sets and category allocations are available in Supplementary Data [see Additional file 1].

The equivalent developmental stages in the developmental series for the CE grown samples (Table 1) were calculated using GenStat^® ^9.0 (VSN, UK) statistical package. Log of expression from the developmental series was linearly interpolated for 32,512 probesets to give a value for every day between 14 and 42 daa. Distance between the 12 CE samples and interpolated developmental samples was defined as square of difference of log expression, summed over all probesets [see Additional file 3]. The daa which gives the minimum distance is the estimate of the equivalent developmental stage for that CE sample. Bootstrapping was used to get an estimate of uncertainty; at each iteration, a sample of size 32,512 was taken at random from the set of probesets, and equivalent daa was recalculated. Two hundred iterations were performed and the distribution in results indicated in Table 1.

### Quantitative RT-PCR

Quantitative reverse transcription PCR was performed in duplicate. Procedures followed were as in [52], with the following modifications: 5 ug of RNA was used for each sample, RNA was DNAse treated before the reverse transcriptase step, and the annealing temperature used was 60 degrees. The data were normalised to an internal control transcript, the probeset for which Ta.2526.1.S1_at (DSS1/SEM1 proteasome subunit family protein) shows consistent expression in array data for all samples presented here. Data are displayed as geometric mean of replicates.

## Authors' contributions

YW contributed to design and carried out the array experimentation; RLP carried out qRT-PCR, AKH did transcription factor analysis. CT-U and KF assisted with material isolation from developmental series and environmental treatment experiments, respectively. SW did the statistical analysis to quantify environmental effects on development. MJG and PRS designed the environmental treatment experiment. ENCM, KJE and PRS together planned the experimental programme. RACM carried out the bioinformatics analyses. PRS, AKH and RACM wrote the manuscript.

## Supplementary Material

Additional file 1 Allocations of probesets to tissue and process categories. Click here for file

Additional file 2 Comparison of clusters in Fig 3 with those of [4,8] and [6]. Click here for file

Additional file 3 Geometric average of expression for all the probesets in each gene cluster shown in Figure 3. Click here for file

Additional file 4 Daa versus distance between interpolated developmental and CE samples. Click here for file

Additional file 5 Phylogenetic tree and sequence alignment of NAC-family TFs. Click here for file

Additional file 6 Phylogenetic tree and sequence alignment of YABBY-family TFs. Click here for file

Additional file 7 Sequence alignment of ARF-family TFs. Click here for file
