# Supplementary material for: Transcriptome analysis of grain development in hexaploid wheat
Source: BMC Genomics. 2008 Mar 6;9:121. doi: 10.1186/1471-2164-9-121 (PMC2292175; doi:10.1186/1471-2164-9-121)

Figure S1. Daa versus geometric average of expression for gene clusters shown in Fig. 3 over the developmental series (left-hand panel) and the four CE samples (control, drought, heat, heat & drought; right-hand panels).

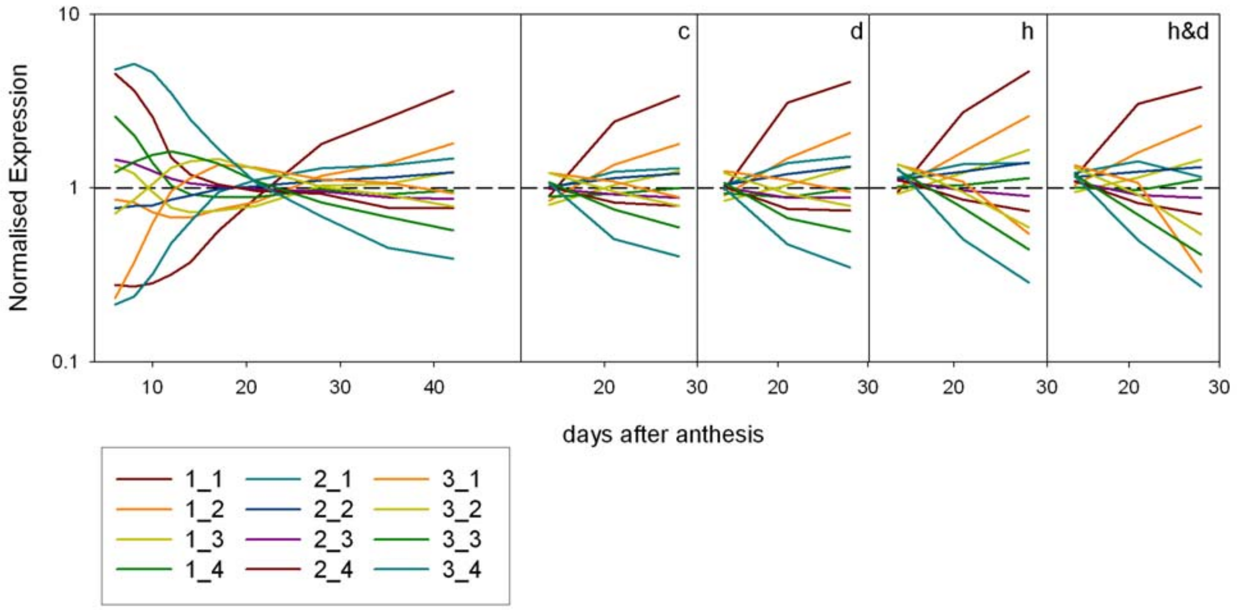

Supplement: Additional file 3 — Geometric average of expression for all the probesets in each gene cluster shown in Figure 3. [file 1471-2164-9-121-S3.pdf]
