# Supplementary material for: Transcriptome analysis of grain development in hexaploid wheat
Source: BMC Genomics. 2008 Mar 6;9:121. doi: 10.1186/1471-2164-9-121 (PMC2292175; doi:10.1186/1471-2164-9-121)
Supplement: Additional file 4 — Daa versus distance between interpolated developmental and CE samples. [file 1471-2164-9-121-S4.pdf]

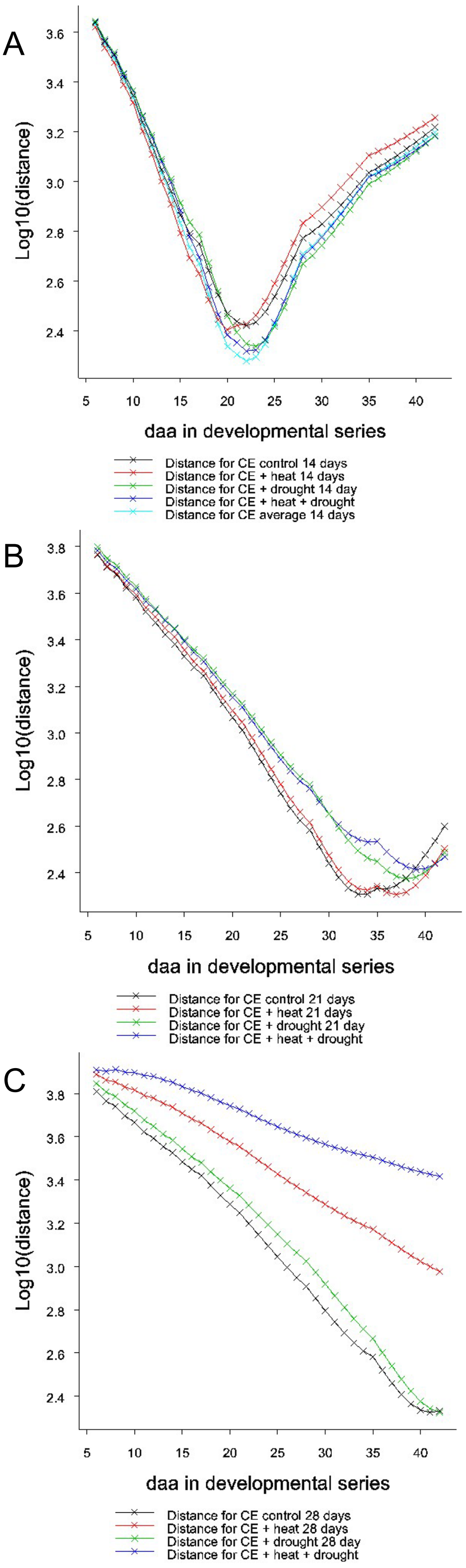

Figure S2. Daa from developmental series versus distance in transcriptome between interpolated developmental series samples and the four CE samples at (A) 14 daa, (B) 21 daa and (C) 28 daa.
