# Supplementary material for: Transcriptome analysis of grain development in hexaploid wheat
Source: BMC Genomics. 2008 Mar 6;9:121. doi: 10.1186/1471-2164-9-121 (PMC2292175; doi:10.1186/1471-2164-9-121)
Supplement: Additional file 5 — Phylogenetic tree and sequence alignment of NAC-family TFs. [file 1471-2164-9-121-S5.pdf]

Figure S3 a Phylogenic tree of NAC proteins. The tree was constructed from the Rice and Arabidopsis sequences from the plnTFDB (Riano-Pachon et.al. 2007), with the addition of the maize genes ZmNRP1 (AY325313), ZmNAC4 (CAH56054), barley TA42485, TA42398, TA6584, TA40870, TA44577, TA41963, wheat TaGrab2 (AJ010832), and probe sets Ta11509.1.S1 (TA82591), Taaffy117676.1.S1 (CA734178), Ta25288.1.S1 (TA80395), Ta37139.1.S1 (TA93469), Ta12286.1.S1 (TA85008). The tree was generated using the maximum likelihood method PHYML (Guindon and Gascuel, 2003). Bootstrap values above 60% are indicated. The tree was constructed from the entire family but only the NAM and NAC1 subclades (Ooka et. al. 2003) are shown. **b**. Amino acid alignment of the five subdomains (A-E) of the NAC DNA binding domain (Ooka *et. al.* 2003). The alignment was generated using CLUSTAL X of the entire family but only sequences in the NAM and NAC1 subclades are shown. Gaps in the sequences and between subdomains are indicated by dashes. Shading is based on amino acid identities and properties.

a

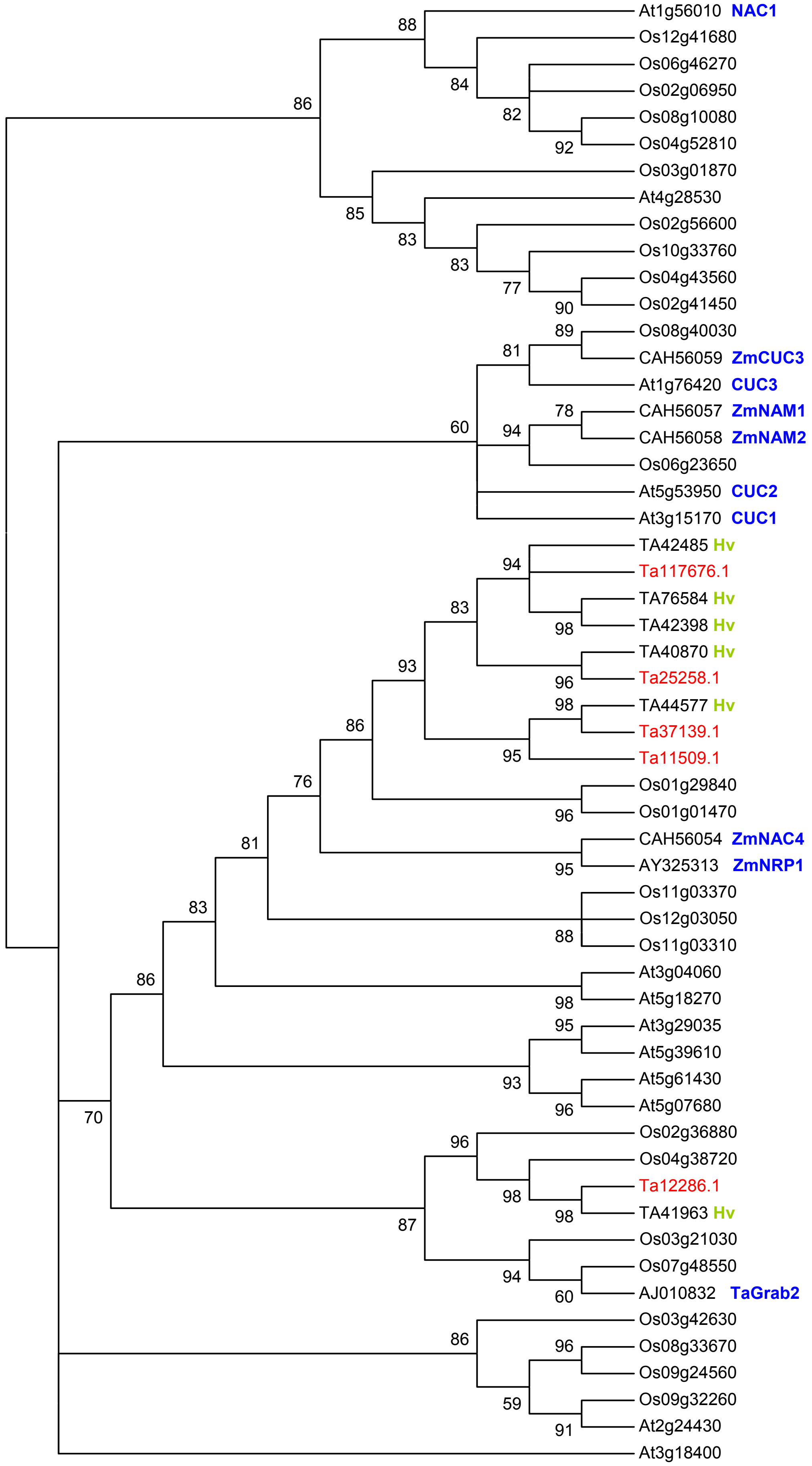
At1g56010 NAC1
Os12g41680
Os06g46270
Os02g06950
Os08g10080
Os04g52810
Os03g01870
At4g28530
Os02g56600
Os10g33760
Os04g43560
Os02g41450
Os08g40030
CAH56059 ZmCUC3
At1g76420 CUC3
CAH56057 ZmNAM1
CAH56058 ZmNAM2
Os06g23650
At5g53950 CUC2
At3g15170 CUC1
TA42485 Hv
Ta117676.1
TA76584 Hv
TA42398 Hv
TA40870 Hv
Ta25258.1
TA44577 Hv
Ta37139.1
Ta11509.1
Os01g29840
Os01g01470
CAH56054 ZmNAC4
AY325313 ZmNRP1
Os11g03370
Os12g03050
Os11g03310
At3g04060
At5g18270
At3g29035
At5g39610
At5g61430
At5g07680
Os02g36880
Os04g38720
Ta12286.1
TA41963 Hv
Os03g21030
Os07g48550
AJ010832 TaGrab2
Os03g42630
Os08g33670
Os09g24560
Os09g32260
At2g24430
At3g18400

b

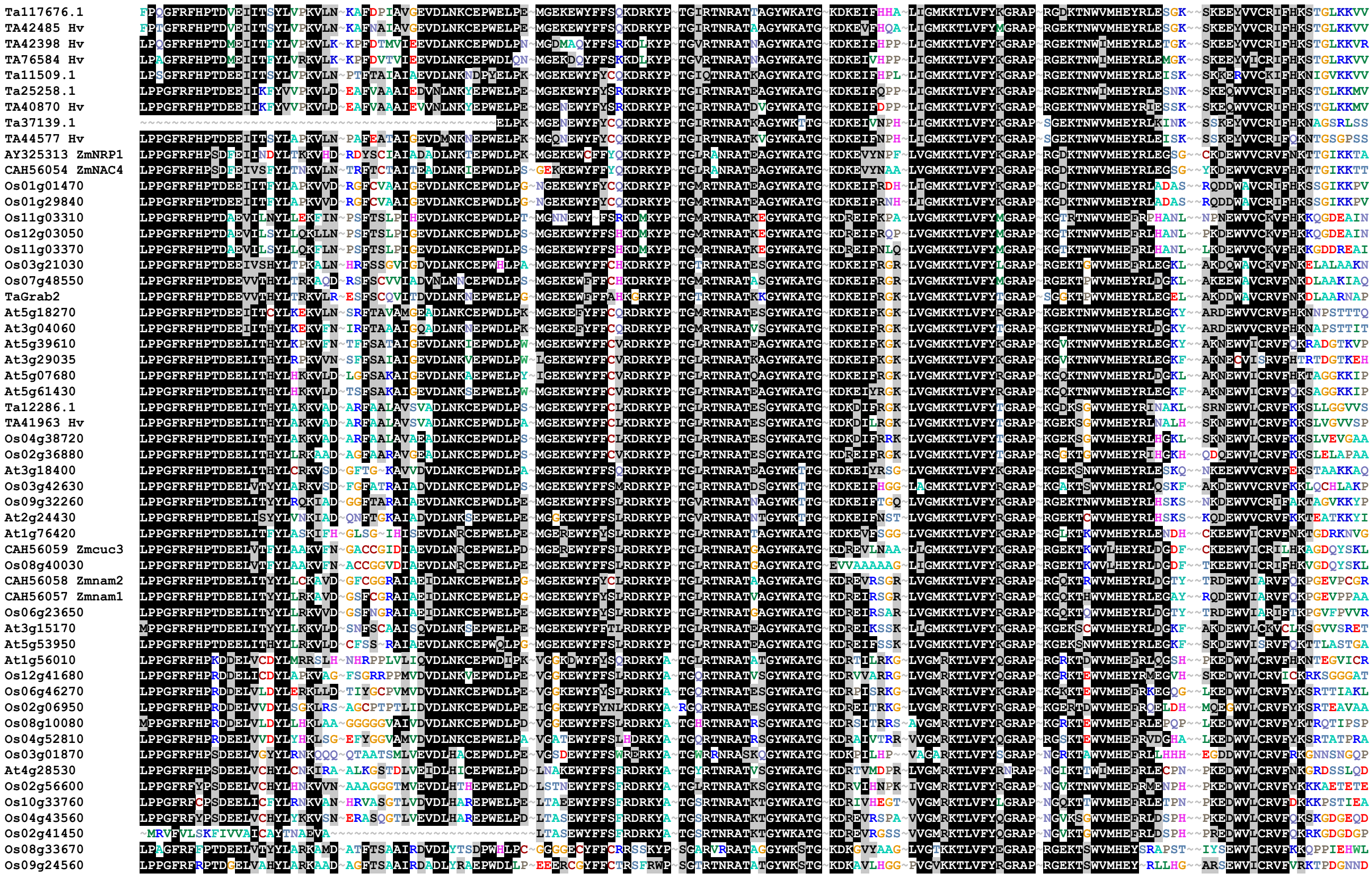

Ta117676.1
TA42485 Hv
TA42398 Hv
TA76584 Hv
Ta11509.1
Ta25258.1
TA40870 Hv
Ta37139.1
TA44577 Hv
AY325313 ZmNRP1
CAH56054 ZmNAC4
Os01g01470
Os01g29840
Os11g03310
Os12g03050
Os11g03370
Os03g21030
Os07g48550
TaGrab2
At5g18270
At3g04060
At5g39610
At3g29035
At5g07680
At5g61430
Ta12286.1
TA41963 Hv
Os04g38720
Os02g36880
At3g18400
Os03g42630
Os09g32260
At2g24430
At1g76420
CAH56059 Zmcuc3
Os08g40030
CAH56058 Zmnam2
CAH56057 Zmnam1
Os06g23650
At3g15170
At5g53950
At1g56010
Os12g41680
Os06g46270
Os02g06950
Os08g10080
Os04g52810
Os03g01870
At4g28530
Os02g56600
Os10g33760
Os04g43560
Os02g41450
Os08g33670
Os09g24560
