# Supplementary material for: Transcriptome analysis of grain development in hexaploid wheat
Source: BMC Genomics. 2008 Mar 6;9:121. doi: 10.1186/1471-2164-9-121 (PMC2292175; doi:10.1186/1471-2164-9-121)
Supplement: Additional file 6 — Phylogenetic tree and sequence alignment of YABBY-family TFs. [file 1471-2164-9-121-S6.pdf]

Figure S4 a. Phylogenic tree of Yabby proteins. The tree was constructed from the rice and Arabidopsis sequences from the plnTFDB (Riano-Pachon et.al. 2007), with the addition of OsYb7 (BAF45808), the barley sequences CA024106, TA34634, TA40171, maize sequences TA207636, TA178808, AY313904, TA190186, TA176465, TA176459 TA220515, TA184239 and wheat proteins corresponding to probe sets Ta4352.1.S1 (AF545436), Ta14101.1.S1(TA64727), Ta7721.1.S1 (TA69349), Ta678.1.S1 (TA90793), Ta7227.1.S1 (CK205871). The tree was generated using the maximum likelihood method PHYML (Guindon and Gascuel 2003). Bootstrap values above 60% are shown.**b.** Amino acid alignment of the zinc finger and YABBY domains generated using CLUSTAL X. Gaps in the sequences and between domains are indicated by dashes.

a

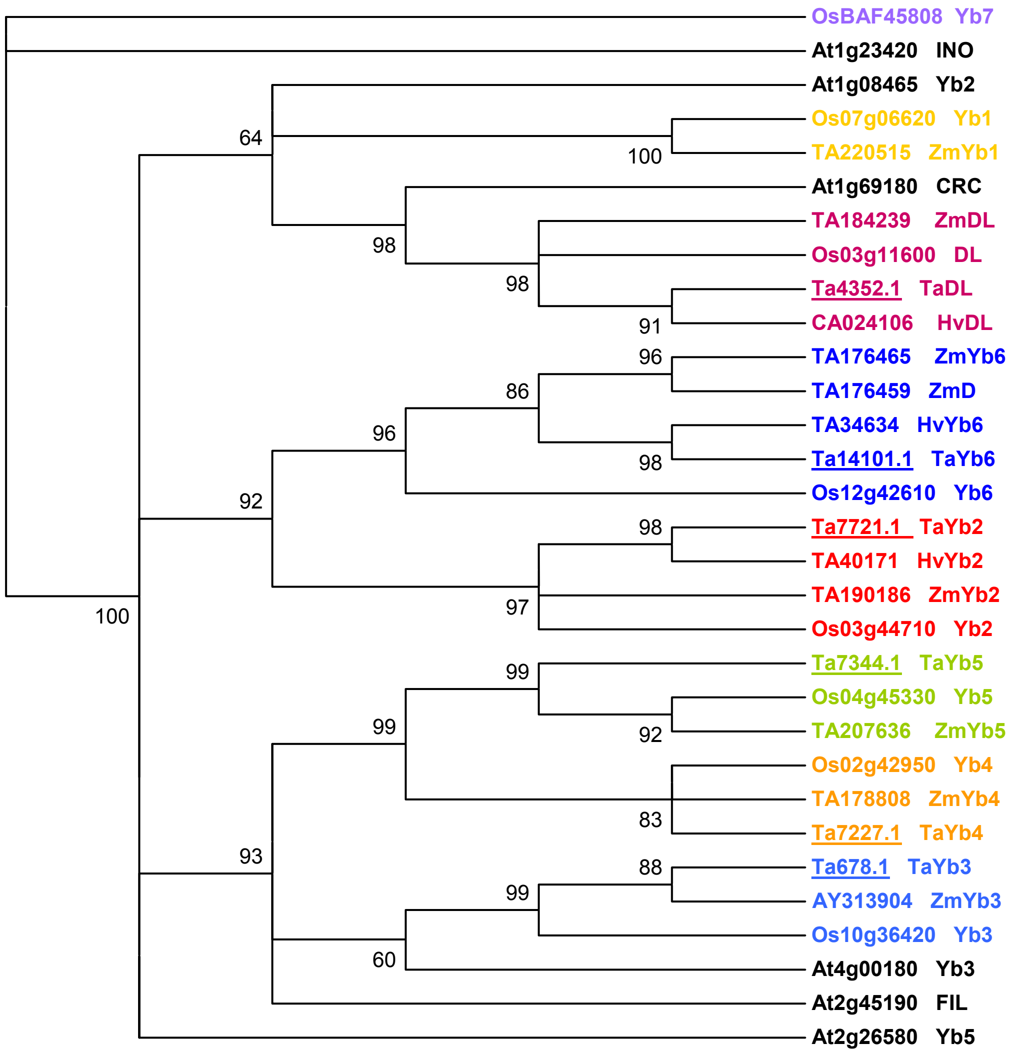

b

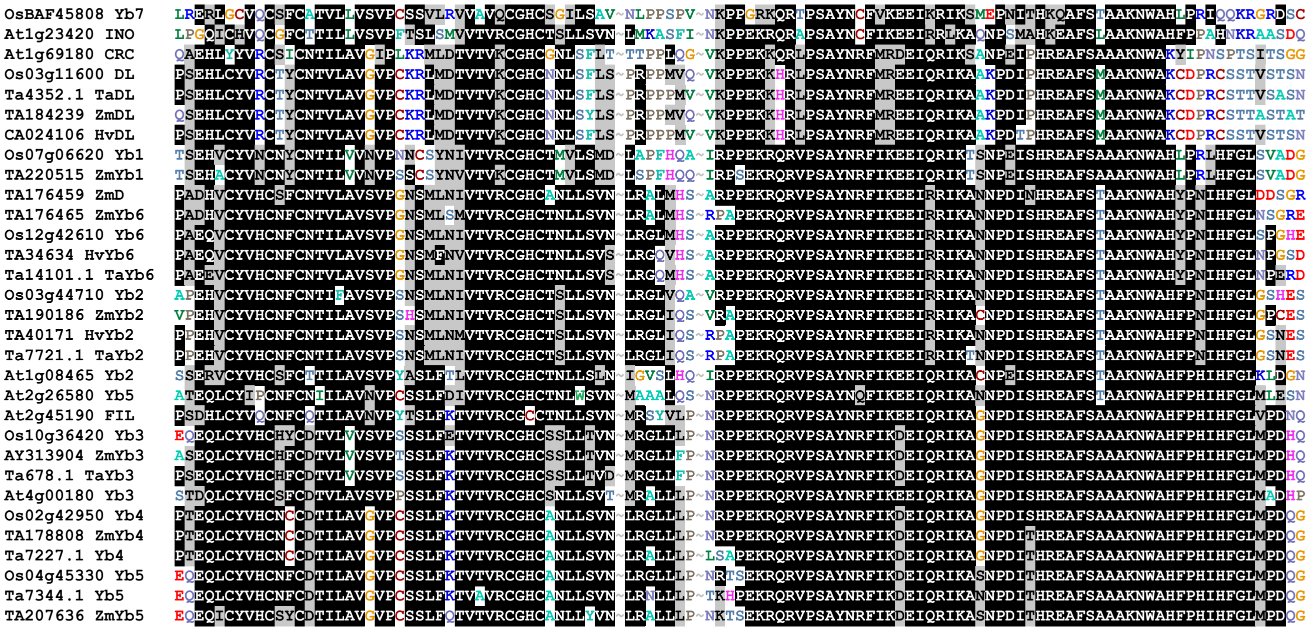

OsBAF45808 Yb7
At1g23420 INO
At1g69180 CRC
Os03g11600 DL
Ta4352.1 TaDL
TA184239 ZmDL
CA024106 HvDL
Os07g06620 Yb1
TA220515 ZmYb1
TA176459 ZmD
TA176465 ZmYb6
Os12g42610 Yb6
TA34634 HvYb6
Ta14101.1 TaYb6
Os03g44710 Yb2
TA190186 ZmYb2
TA40171 HvYb2
Ta7721.1 TaYb2
At1g08465 Yb2
At2g26580 Yb5
At2g45190 FIL
Os10g36420 Yb3
AY313904 ZmYb3
Ta678.1 TaYb3
At4g00180 Yb3
Os02g42950 Yb4
TA178808 ZmYb4
Ta7227.1 Yb4
Os04g45330 Yb5
Ta7344.1 Yb5
TA207636 ZmYb5
