# Supplementary material for: Transcriptome analysis of grain development in hexaploid wheat
Source: BMC Genomics. 2008 Mar 6;9:121. doi: 10.1186/1471-2164-9-121 (PMC2292175; doi:10.1186/1471-2164-9-121)
Supplement: Additional file 7 — Sequence alignment of ARF-family TFs. [file 1471-2164-9-121-S7.pdf]

Figure S5. Alignment using the CLUSTAL X program of wheat ARF proteins corresponding to the probe sets Ta7431.1.S1 (TA76217), Ta13246.1.S1 (TA76218), and Ta2593.2.S1 (TA54498) with rice and Arabidopsis proteins from the plnTFDB (Riano-Pachon et. al. 2007) showing the highest BLAST scores. The upper group of proteins correspond to members of subgroup Class III and the lower group to subclass Ia (Wang *et. al*. 2007). Shading is based on amino acid identities and properties.

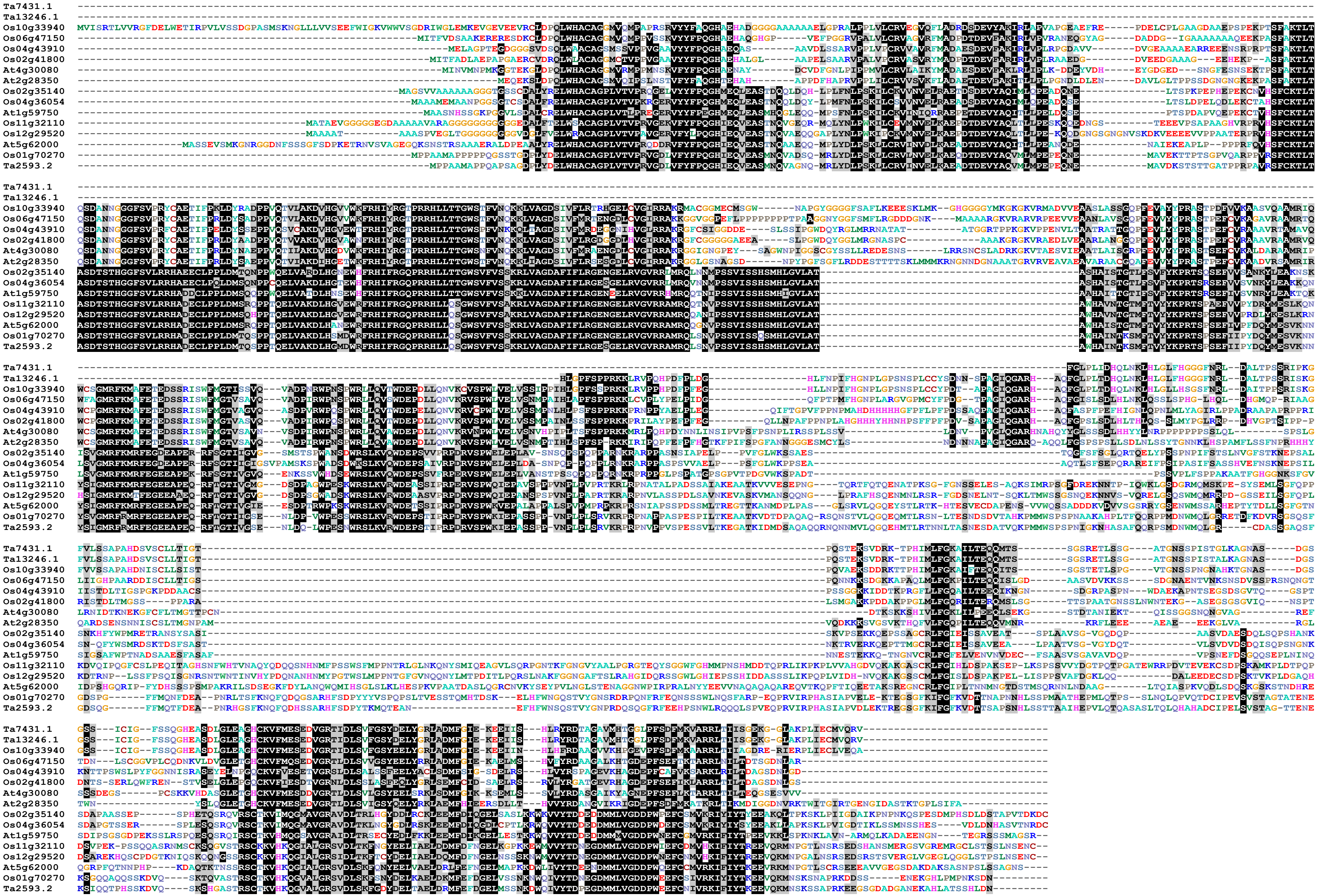
Ta7431.1
Ta13246.1
Os10g33940
Os06g47150
Os04g43910
Os02g41800
At4g30080
At2g28350
Os02g35140
Os04g36054
At1g59750
Os11g32110
Os12g29520
At5g62000
Os01g70270
Ta2593.2
